# Honeybee Sentience: Scientific Evidence and Implications for EU Animal Welfare Policy

**DOI:** 10.3390/vetsci12070661

**Published:** 2025-07-12

**Authors:** Roberto Bava, Giovanni Formato, Giovanna Liguori, Fabio Castagna

**Affiliations:** 1 Department of Health Sciences, University of Catanzaro Magna Græcia, 88100 Catanzaro, Italy; roberto.bava@unicz.it (R.B.); fabiocastagna@unicz.it (F.C.); 2 Apiculture Laboratory, Istituto Zooprofilattico Sperimentale del Lazio e della Toscana “M. Aleandri”, Via Appia Nuova 1411, 00178 Rome, Italy; giovanni.formato@izslt.it; 3 Local Health Autorithy (ASL) Foggia, 71121 Foggia, Italy

**Keywords:** animal behavior, honeybee welfare, invertebrate cognition, EU legislation

## Abstract

The growing recognition of animal sentience has led to notable progress in European Union animal welfare legislation. However, a significant inconsistency remains: while mammals, birds, and cephalopods are legally protected as sentient beings, honeybees (*Apis mellifera*)—despite robust scientific evidence of their cognitive, emotional, and sensory complexity—are excluded from such protections. This manuscript examines, from an interdisciplinary perspective, the divergence between emerging evidence on invertebrate sentience and current EU legal frameworks. Honeybees and cephalopods serve as comparative case studies to assess inconsistencies in the criteria for legal recognition of sentience. Findings increasingly confirm that honeybees exhibit advanced cognitive functions, emotional states, and behavioral flexibility comparable to those of legally protected vertebrates. Their omission from welfare legislation lacks scientific justification and raises ethical and ecological concerns, especially given their central role in pollination and ecosystem stability. In general, we advocate for the inclusion of *Apis mellifera* in EU animal welfare policy. However, we are aware that there are also critical views on their introduction, which we address in a dedicated paragraph of the manuscript. For this reason, we advocate a gradual and evidence-based approach, guided by a permanent observatory, which could ensure that legislation evolves in parallel with scientific understanding, promoting ethical consistency, sustainable agriculture, and integrated health under the One Health framework. This approach would meet the concerns of consumers who consider well-being and respect for the environment as essential principles of breeding, and who carefully choose products from animals raised with systems that respect welfare, with indisputable economic advantages for the beekeeper.

## Simple Summary

This manuscript highlights a critical gap in European Union animal welfare legislation, which currently recognizes mammals, birds, and cephalopods as sentient beings, while excluding honeybees (*Apis mellifera*) despite growing scientific consensus on their complex cognitive, emotional, and sensory capacities, and even though the New York Declaration on Animal Consciousness in 2024 acknowledged the realistic possibility of consciousness in invertebrates, including *Apis mellifera*, emphasizing the importance of considering their welfare based on scientific evidence. This analysis compares honeybees and cephalopods to expose inconsistencies in the legal acknowledgment of invertebrate sentience. The evidence demonstrates that honeybees possess behavioral and neurological traits akin to those of protected vertebrates, making their exclusion from legal safeguards both scientifically unfounded and ethically problematic. Given their vital role in pollination and ecosystem balance, the neglect of bee welfare has a direct impact in terms of One Welfare, posing broader ecological and agricultural risks. The authors propose a gradual, science-driven expansion of existing welfare policies, supported by a permanent observatory to ensure that legal standards remain aligned with evolving scientific knowledge. Such an approach would not only enhance the coherence of EU legislation but also support sustainable food systems and public health within the integrative One Health paradigm.

## 1. Introduction

In recent decades, increasing awareness of animal welfare and its implications for food safety and public health has prompted a reconsideration of livestock farming policies within the European Union (EU). Scientific evidence demonstrates that inadequate farming conditions not only compromise animal health and welfare but also negatively impact the quality of animal-derived food products, increasing the risk of zoonotic diseases [1,2,3]. Concurrently, European consumers with moderate to high food literacy have shown growing interest in production systems that prioritize animal well-being, associating such practices with higher product quality [4].

Among producers, the concept of improving farm animal welfare is increasingly seen not as a financial burden, but as a market opportunity capable of justifying fair pricing through alignment with consumer values. This shift is gradually extending to the beekeeping sector, where quality is no longer defined solely by the organoleptic properties of honey and hive products, but also by the living conditions of pollinating insects and their environmental sustainability.

Historically, many beekeepers viewed welfare practices—such as minimizing transport stress, avoiding aggressive chemical treatments, and preserving biodiversity—as added costs that reduced competitiveness. However, this perception is evolving. A growing number of producers now recognize that ensuring bee welfare is not only an ethical commitment but also a commercially advantageous strategy. This trend is reinforced by the success of certifications such as that for organic beekeeping [5] and animal welfare labels applied to insects, which are increasingly present in supermarkets and specialty shops. These certifications represent more than quality marks; they attest to the respect for bees’ natural life cycles, reduced handling stress, and optimal conditions for colony development. The visibility of such practices is amplified by e-commerce platforms and ethical consumer networks, which selectively reward companies that adopt certified, bee-friendly protocols.

At present, major farmed animal species are regulated under EU animal welfare legislation. Nevertheless, despite significant advancements in neuroethology and cognitive science, which have shown that some invertebrates (such as honeybees (*Apis mellifera*), crustaceans, and cephalopods) exhibit complex cognitive and emotional capacities comparable to those of protected vertebrates [6,7], a regulatory gap persists. Cephalopods are currently recognized as sentient beings and included under EU welfare legislation, while honeybees, despite growing evidence of their sentience, remain excluded. It is accepted that neural architecture alone is not predictive of sentience: diverse structures (e.g., the decentralized brain of octopuses) can support analogous cognitive functions. This inconsistency generates legal paradoxes: while octopuses are protected, bees, despite exhibiting comparable cognitive abilities, are not.

Even the New York Declaration on Animal Consciousness, a document endorsed by numerous university scholars and researchers from around the world, in 2024 acknowledged the realistic possibility of consciousness in invertebrates, including *Apis mellifera*, emphasizing the importance of considering their welfare based on scientific evidence [8].

This position paper synthesizes evidence from neurobiology, ethology, and EU policy to advocate for the inclusion of honeybees (*Apis mellifera*) under animal welfare legislation. By examining the disconnect between scientific knowledge and EU policy and arguing that the absence of legal protection for ecologically and economically vital species such as *Apis mellifera* is no longer scientifically tenable, the manuscript sheds new light on this issue. The central hypothesis is that the current exclusion of honeybees from EU welfare legislation lacks robust scientific justification, as converging evidence indicates that bees and cephalopods share integrative neural pathways (e.g., an opioid-like system in bees), exhibit complex behaviors (such as associative learning and symbolic communication), and display emotional responses including optimism/pessimism biases [9,10]. Through an interdisciplinary investigation of scientific literature, the paper highlights functional convergences in learning, emotional states, and nociception between bees and other protected species, revealing that what is often treated merely as a productive sector conceals significant ethical, ecological, and economic contradictions.

Although there are criticisms that support the opposite point of view, analyzed in a specific paragraph of the manuscript, aligning EU regulations with current evidence on honeybee cognition and emotion seems to be a scientific and ethical necessity, not only to mitigate stress-related disorders but also to ensure colony health.

Formal recognition of the sentience of bees would require ethical review protocols for invasive procedures (e.g., hemolymph sampling); guidelines to reduce stress in transport (maximum a few hundred km per day, also in relation to ambient temperature and humidity); hive inspection protocols for minimal brood disruption (e.g., ≤5 min/hive during peak activity hours) to reduce colony stress; alternatives to colony sacrifice in disease control (e.g., quarantine protocols). These measures could address welfare issues.

A phased reform—grounded in science and stakeholder dialogue—is needed to translate evidence into feasible standards. This reform could be particularly reasonable given that bees are intensively farmed for food products (honey and pollen) and substances used in pharmaceutical and cosmetic industries (propolis, royal jelly, and venom), whose safety may ultimately impact human health. The evidence brought to attention, though not conclusive, warrants a precautionary approach under One Health principles. We propose three key actions: (1) phased inclusion of honeybees in Directive 2010/63/EU; (2) a sentience observatory to review emerging evidence biannually; (3) stakeholder consultations to balance welfare with apicultural needs.

In this manuscript, we adopt the operational definitions summarized in Table 1:

## 2. Reframing Sentience: Animal Welfare and the Regulatory Oversight of Invertebrates in the EU

Animal sentience, defined as the capacity of an organism to experience subjective states with affective valence, such as pain, pleasure, or stress, must be clearly distinguished from nociception, the unconscious physiological response to potentially harmful stimuli [11].

While nociception is widespread across the animal kingdom, even present in organisms lacking a central nervous system (e.g., jellyfish), sentience implies a higher level of complexity [12,13]. It requires not only stimulus reception but also conscious processing. In vertebrates, this is typically associated with cortical regions, whereas in invertebrates, functionally analogous structures—such as the mushroom bodies in insects—support similar cognitive functions. Key features include: (1) affective memory, which enables animals to modify future behavior based on past experiences; (2) motivational trade-offs, or the ability to weigh positive and negative stimuli in decision-making; (3) conscious awareness, capabilities that transform physiological responses into subjective experiences with ethical ramifications. This distinction underpins modern animal welfare frameworks: whereas nociception justifies basic precautions (e.g., avoiding tissue damage), sentience demands recognition of an animal’s right to protection from avoidable suffering [14]. Historically, however, legal and ethical standards have struggled to align with this scientific nuance.

The pivotal shift began in 1964 with Ruth Harrison’s *Animal Machines* [15], which exposed the welfare deficiencies in intensive farming and spurred the Brambell Committee [16] to establish the five freedoms criteria emphasizing freedom from hunger, pain, discomfort, and fear, alongside the ability to express natural behaviors. While these principles laid the foundation for modern welfare standards, their application was explicitly limited to vertebrates, despite early empirical evidence of consciousness in invertebrates, such as von Frisch’s behavioral studies on bees. This taxonomic bias persisted even as welfare science evolved to prioritize individual adaptability and quality of life [1,17,18]. The Treaty of Amsterdam (1997) [19] marked progress by legally recognizing animals as sentient under EU law, yet its operational focus remained on vertebrates. This recognition was reaffirmed in the Treaty of Lisbon, with Article 13 of the Treaty on the Functioning of the European Union [20] mandating that Member States consider animal welfare in agriculture, research, and transport.

However, the absence of a clear operational definition of sentience has led to significant regulatory gaps. Notably, invertebrates, despite mounting evidence of complex cognition and emotion, are generally excluded from protections, with the exception of cephalopods as defined in Directive 2010/63/EU [21]. This taxonomic approach, which prioritizes biological classification (vertebrates vs. invertebrates) over functional and neurobehavioral criteria (e.g., presence of nociceptive pathways, cognitive markers), is increasingly outdated in light of emerging empirical data. As such, the concept of animal welfare must be seen as dynamic and responsive to advances in neuroscience, ethology, and behavioral biology.

To reconcile science with regulation, a novel functional welfare definition is proposed for managed *Apis mellifera:* “a balanced and dynamic state, as natural as possible, where the beehive superorganism and each individual bee have the freedom to express their roles and preferences, meet their fundamental needs, and adapt positively to variable external stressors without enduring unnecessary suffering” [22]. This definition encapsulates the core principles of honey bee welfare, recognizing both individual and colony-level requirements while highlighting the importance of adaptability and natural living conditions.

## 3. Reevaluation of Invertebrate Sentience: Neurobiological Evidence and Ethical Implications

The discrepancy between scientific evidence and traditional conceptions becomes particularly evident when examining the neural structures of invertebrates. Classical approaches often link cognitive complexity and sentience exclusively to brain size or centralization degree, assumptions now debunked anatomically and physiologically. Recent comparative studies demonstrate that cognitive complexity depends on two key factors: synaptic density and functional specialization. For instance, the honeybee brain, with approximately one million neurons [23], exhibits connectivity comparable to vertebrates, highlighting the importance of synaptic density [24,25,26]. Functional specialization is evident in the mushroom bodies of insects, which show striking parallels to the associative cortex of vertebrates in their role in multisensory integration and learning [27].

Invertebrate nervous systems have evolved into two primary strategies: centralized and cognitive forms, as seen in cephalopods and some social insects, and more decentralized yet functionally effective organizations, characteristic of crustaceans and bees. This diversity reflects the vast morphological and behavioral variety among invertebrates and a surprising evolutionary convergence toward neural solutions supporting advanced cognitive abilities.

Cephalopods, particularly the common octopus (*Octopus vulgaris*), present a unique case among invertebrates. Despite evolutionary distance from vertebrates, their brains exhibit sophisticated and highly specialized organization. The vertical, superior, and optic lobes of octopuses show “low-amplitude fast” neural activity similar (but not homologous) to the mammalian thalamocortical complex [23,24], indicating evolutionary convergence [28,29].

The inclusion of cephalopods in EU Directive 2010/63/EU marks a pioneering recognition of invertebrate sentience based on neurofunctional criteria rather than taxonomy [21]. However, this approach reveals an inconsistency: if a distributed nervous system like that of octopuses can support subjective experiences, excluding other invertebrates, such as bees, exhibiting similar cognitive abilities—associative learning, long-term memory, and behavioral flexibility, albeit with different neural architectures—becomes arbitrary.

Bees, as social insects, possess a fragmented yet efficient nervous system. Their brains, with one million neurons, support complex behaviors: associative learning, spatial navigation, symbolic communication (e.g., the famous waggle dance), and even individual recognition. The anatomy of their nervous system includes three main areas: the brain (processing visual and olfactory information), the gnathocerebrum (controlling feeding functions), and the ventral ganglion chain (motor coordination) [30,31]. The presence of mushroom bodies, higher associative centers, and the sophisticated organization of the nervous system enable colonies to function as true “superorganisms”, where cooperation and communication occur through highly coordinated chemical, visual, and mechanical signals [32].

The discovery of alpha oscillations in bee brains, similar to those associated in vertebrates with complex cognitive functions like attention and memory, represents a surprising breakthrough [33]. Popov and Szyszka (2020) reveal that alpha oscillations in honeybee brains, comparable to those in humans, govern interhemispheric spike timing coordination [33]. This suggests conserved mechanisms for neural synchronization, hinting at functional parallels (e.g., attention-like processes) despite evolutionary distance [33]. This finding, coupled with the sophisticated neural structure of bees that in some aspects matches that of cephalopods and vertebrates, challenges traditional hierarchical models based on taxonomy or brain size.

The comparative analysis of neural systems across taxa necessitates careful distinction between evolutionary homology (traits inherited from a common ancestor) and functional convergence (independent evolution of similar traits). While vertebrates and invertebrates share no recent ancestry in brain structures, striking functional analogies exist. For instance, the mushroom bodies of insects and the vertebrate pallium both support associative learning, yet arise from developmentally distinct tissues [29]. Similarly, octopod vertical lobes and mammalian cortices exhibit comparable information-processing roles despite lacking homologous origins [28,34,35]. These convergences suggest that natural selection has repeatedly favored integrated neural architectures for complex behavior, irrespective of phylogenetic starting points. Crucially, welfare-relevant cognitive capacities (e.g., pain processing, emotional states) need not depend on homology with vertebrate systems to merit ethical consideration—a principle already acknowledged in the EU’s inclusion of cephalopods under Directive 2010/63/EU.

## 4. Consolidated Neural Evidence and Global Workspace Theory

While anatomical analysis is fundamental, it alone is insufficient to demonstrate sentience. Contemporary cognitive models provide decisive interpretative tools for understanding consciousness across diverse neural architectures.

Two frameworks are particularly relevant for evaluating invertebrate consciousness.

The first is the global workspace theory, originally developed for vertebrates, but applicable to invertebrates like cephalopods and bees [36,37]. This model posits that consciousness emerges when specialized neural modules integrate information into a “global workspace” accessible across the brain. In octopuses, despite their decentralized nervous system (with 60% of neurons distributed in the arms), a vertical lobe integrates multisensory information for complex behaviors [38,39]. Similarly in bees, their mushroom bodies act as integration centers, processing visual and olfactory inputs for flexible decision-making [40]. These structures, though anatomically different from the mammalian cortex, fulfill analogous functions in creating unified sensory experiences and coordinating adaptive responses. The theory demonstrates how conscious awareness can emerge from distributed processing without requiring a centralized, mammalian-like brain.

The second is the integrative threshold model developed by Barron and Klein (2016) [41] that complements this framework, proposing that consciousness evolved as a tool to evaluate ambiguous stimuli in ecologically critical contexts. According to this model, the primary function of consciousness is to integrate diverse sensory inputs, internal states, and memory to generate unified representations of the organism’s relationship to its environment. In bees, this capacity is confirmed in cognitively sophisticated behaviors: bumblebees (*Bombus terrestris*) demonstrate an “optimistic bias” when interpreting ambiguous stimuli after receiving a reward [42]. This behavior is modulated by dopamine, similarly to that in vertebrates. Similarly, honeybees (*Apis mellifera*) build internal spatial representations guiding complex decisions, from orientation to floral choice [43]. These theoretical frameworks converge in identifying three fundamental pillars of consciousness that manifest across diverse species: (1) multimodal sensory integration; (2) behavioral flexibility beyond simple stimulus-response patterns; (3) and neurochemical modulation of subjective states.

The neurobiological evidence for each of these pillars in honeybees includes: multimodal integration (mushroom bodies receive and integrate visual, olfactory, and mechanosensory inputs to create unified representations of environmental stimuli); behavioral flexibility (navigation using cognitive maps, rule learning, abstract concept formation); neurochemical modulation (conserved systems (dopaminergic, serotonergic, octopaminergic) that regulate behavior, learning, and affective states). The high synaptic density [44] and neural specialization of hymenopteran mushroom bodies [27] further weaken the traditional distinction between “sentient” vertebrates and “merely reactive” invertebrates, suggesting instead a continuum of cognitive abilities supported by different neural architectures. These theories find practical application in the study of pain perception in bees, where anatomical, physiological, and behavioral evidence converge, raising urgent ethical questions about current regulatory disparities.

## 5. Cognition, Communication, and Emotional States in Honeybees (*Apis mellifera*): Insights into Invertebrate Intelligence

Several studies in animal cognition have uncovered unexpectedly sophisticated abilities in invertebrates. Early discoveries have revealed evidence of social learning and problem-solving in bees. Huber observed bees modifying hive architecture to accommodate unforeseen structural constraints, implying a degree of design flexibility inconsistent with reflexive behavior [45]. Another historical and well-known example is the waggle dance of honeybees (*Apis mellifera*), a spatially encoded communication system first described by Karl von Frisch [46]. While the dance’s basic structure is innate, its execution is adaptively modulated by environmental cues: distance calibration via optic flow [47], and angle adjustment to gravity changes. This plasticity—paired with individual variability in associative learning (e.g., color-reward discrimination) [48] demonstrates behavioral flexibility beyond rigid instinct, meeting criteria for learned plasticity in welfare science. The system exhibits proto-linguistic properties, including arbitrariness of signs, displaced reference, and communicative intentionality.

The waggle dance reflects a deeper integration of spatial knowledge. Harmonic radar tracking studies reveal that bees navigate using an allocentric, map-like memory [49]. When displaced to unfamiliar locations, foragers perform novel shortcuts between known sites (e.g., hive and feeder), suggesting cognitive flexibility beyond simple path integration.

Investigations showed bees can communicate information about past foraging sites even without immediate necessity, indicating long-term mental representations [50]. Menzel and Giurfa (2001) demonstrated contextual learning and rule-based flexibility, mediated by mushroom bodies [24].

Similarly, Loukola et al. (2017) found that bumblebees can imitate conspecifics to solve novel tasks, such as moving a ball to a target, and improve upon observed techniques [51].

Bees also learn abstract relational rules, “same” and “different”, and apply them to novel stimuli [52], with neurobiological parallels in alpha oscillations [33]. As highlighted by Hussaini et al. (2009), these findings imply that bees can rework information during sleep, consolidating complex memories such as spatial or extinction memories, with an active role of sleep comparable to that of mammals [53]. Further supporting the complexity of honeybee subjective states, studies on sleep dynamics reveal striking parallels with vertebrates. Foragers exhibit three distinct sleep stages (FS (flexible stage), SS (slow-wave stage), and TS (deep sleep stage)), characterized by progressive muscle relaxation, reduced antennal motility, and elevated response thresholds—during deep sleep (TS), stimuli must be 10,000× more intense to elicit a response compared to wakefulness [54]. Sleep-deprived bees show rebound effects, compensating for lost rest through prolonged sleep periods, a hallmark of homeostatic regulation [55]. Preliminary evidence also suggests rhythmic brain activity in the mushroom bodies during sleep, akin to memory-consolidating oscillations in vertebrates [55]. These findings align with the integrative threshold model, implying that sleep’s role in neural restoration and memory consolidation may reflect a form of subjective experience [41].

Emotional processing in bees further supports their cognitive complexity. Bateson et al. (2011) documented stress-induced pessimistic biases in honeybees, while Solvi et al. (2016) revealed dopamine-mediated positive states in bumblebees [42,56]. Bees subjected to brief agitation were less likely to investigate ambiguous cues, indicating altered affective expectations [56]. These responses persist beyond immediate stimulus, suggesting enduring internal states.

Neurochemical parallels with vertebrates include dopaminergic, serotonergic, and octopaminergic systems [57,58,59]. For example, caffeine in floral nectar enhances long-term memory consolidation in honeybees, improving their retention of reward associations, mirroring known nootropic effects in vertebrates [60].

Chronic stress in invertebrates induces neuroendocrine responses analogous to those of vertebrates, including elevated octopamine levels and long-term behavioral changes [61]. Particularly, chronic stress impairs immune function and reproduction in vertebrates and invertebrates, highlighting conserved neuroendocrine-immune interactions [62,63]. These parallels have practical implications for apiculture, where unmanaged transport and handling of colonies can induce chronic stress and compromise survival, underscoring the importance of welfare protocols.

Individual variation in behavior further enriches our understanding of bee cognition. Consistent inter-individual differences in boldness have been documented in honeybees, with some individuals displaying risk-prone or risk-averse behavior [64]. Bold individuals, often identified as “scout bees” typically constitute a minority of the foraging force (5–25%) but disproportionately influence colony foraging strategies [65]. Molecular studies reveal differential expression of genes related to neurotransmitter systems in scout bees, particularly involving dopamine pathways, suggesting a neurobiological basis for these behavioral differences [64,66].

During nest-site selection, colonies achieve consensus through decentralized processes, evaluating criteria like cavity size (ideal: 30–60 L) and entrance height (~3 m above ground), with the best site eventually winning through a quorum-based system [65]. This collective decision-making demonstrates sophisticated group cognition.

Finally, honeybees perform advanced cognitive tasks including facial recognition [67] and basic arithmetic operations [68], challenging assumptions about brain size and cognitive capacity.

## 6. Pain Perception in Bees: Anatomical and Behavioral Evidence

Bees possess a complex nervous system, including mechanosensory and chemosensory structures that process a wide array of environmental inputs. While honeybees exhibit robust nociceptive responses to injury (e.g., prolonged grooming of wounds, opioid-modulated avoidance learning [69,70]), we emphasize these reflect protective reflexes, not conclusive evidence of phenomenological pain akin to vertebrates. Crucially, their demonstrated capacity for motivational trade-offs [69] operationally aligns with the sentience criteria applied to cephalopods in Directive 2010/63/EU, despite unresolved questions about subjective experience. The question of whether bees truly feel pain, not just react to harm, has major implications for both science and ethics. Bees detect damage through specialized nerve cells that fire at dangerous temperatures or pressures, much like our own pain sensors, but the real mystery is whether they experience suffering the way vertebrates do. Bees have sophisticated nervous systems with (1) damage detectors that respond to threats (like heat over 45 °C); (2) behavioral responses to injury (limb guarding, obsessive wound-grooming); and (3) neurochemicals similar to our natural painkillers (enkephalins) [71,72,73]. However, key differences stand out: (1) their nerve signals stop short of higher brain areas linked to emotional processing in mammals; (2) they lack the classic opioid receptors that mediate pain relief in vertebrates; (3) even their morphine preference does not prove they feel relief, just attraction [70].

While conclusive evidence of pain phenomenology in bees remains debated, their injury responses (e.g., prolonged grooming of wounds) and stress-induced cognitive impairments (e.g., disrupted navigation) warrant ethical consideration.

This precautionary approach balances scientific uncertainty with ethical responsibility, because even without definitive proof of pain, their sophisticated biology demands our respect. Taken together, anatomical, pharmacological, and behavioral data support the hypothesis that bees may experience a form of pain encompassing both sensory and affective dimensions. While these findings suggest bees may experience pain-like states, we note this interpretation remains debated. Nevertheless, a precautionary approach is justified given their ecological importance and behavioral complexity.

Therefore, it should minimize transport stress, monitored behavior for distress and considered anesthesia in invasive research.

The growing evidence of honeybee sentience does not just challenge scientific paradigms: it demands concrete changes in how we study and manage these insects. If the EU were to formally recognize bee sentience, as we propose, this would have immediate practical repercussions across multiple domains. Unlike vertebrates, honeybee welfare requires colony-level protocols.

Drawing lessons from cephalopod welfare protocols [74], several critical adjustments needed can be identified: (1) research involving invasive procedures, like hemolymph sampling or forced starvation assays, would require proper ethical review, similar to vertebrate studies; (2) migratory beekeeping operations might need to adopt daily transport limits (under 200 km/day with temperature control), while hive inspections during research should be time-limited (≤5 min during peak activity hours) to minimize colony disruption; (3) disease monitoring could shift toward non-lethal methods like PCR testing, rather than whole-colony destruction. For unavoidable euthanasia, rapid freezing after anesthesia should replace current methods like soapy water immersion.

While these changes might increase some operational costs initially, they mirror the ethical progression we have seen in vertebrate research. Legislative inclusion need not be binary. The EU’s precedent with cephalopods (Directive 2010/63/EU) demonstrates how evidence-based thresholds can guide protections without indiscriminate overextension. For honeybees, we propose three inclusion criteria: commercial exploitation (e.g., migratory beekeeping, royal jelly production) vs. wild colonies; cognitive/affective markers, intended as demonstrated capacities for associative learning, stress-induced biases, and pain-like responses; human health linkages: colony stress as a risk factor for diseases (e.g., American foulbrood) and food safety (e.g., antibiotic overuse). Improving welfare practices may enhance bee health and productivity, while increasing the reliability and reproducibility of scientific outcomes derived from honeybee studies. Table 2 below summarizes the concepts clarified in the manuscript, putting special emphasis on the possible similarities between bees and cephalopods and the resulting legislative gaps.

## 7. Objections to Honey Bee Consciousness: A Comprehensive Critical Analysis

While some researchers argue convincingly that the behavioral complexity of bees and striking neurobiological analogies to more complex organisms suggest the existence of some form of consciousness [10,75], a growing body of objections challenges this hypothesis on empirical, theoretical, and evolutionary grounds. These criticisms articulate around four fundamental pillars: neuroanatomical limitations, evolutionary implausibility, alternative mechanistic explanations for complex behaviors, and epistemological constraints in attributing consciousness.

According to critics, the bee brain, however sophisticated within its reduced dimensions, simply does not possess the structural prerequisites necessary to host this interior spectacle. With one million neurons and without any equivalent of the brain structures that in vertebrates seem fundamental for consciousness, such as thalamocortical circuits or the claustrum, the insect brain might be simply incapable of generating unified phenomenological states [76]. The mushroom bodies, those elegant structures that in bees mediate learning and memory, operate through modular, feedforward circuits optimized for efficiency—a design that, according to critics, would preclude the global integration and recurrent processing theorized as necessary for consciousness [77]. Without neural substrates for metacognition or affective evaluation, bee behaviors might be completely explained by non-conscious computations [78,79].

From an adaptive perspective, opponents raise a fundamental question: why would evolution have endowed bees with consciousness, considering the metabolic and computational costs this would entail? Abbate (2022) [80] observes that bees show highly stereotyped, task-specific behaviors—from the waggle dance that communicates flower locations to hive coordination guided by pheromones—that might require only innate algorithms rather than flexible, experience-driven decision-making. Unlike vertebrates, which face unpredictable predation and complex social dynamics, bees operate in environments where pre-programmed routines such as phototaxis and circadian foraging are optimally efficient. It is significant, moreover, that bees lack those behavioral markers linked to conscious suffering that are observed in other animals: learned helplessness or trade-offs between harm avoidance and competing motivations [81]. This suggests that their responses to damage are reflexive nociception, not true pain [82]. When DeGrazia (2020) [75] argues for the motivational benefits of sentience, critics respond with simulations showing how reinforcement learning in robots achieves goal-directed behaviors without phenomenological states [79].

Bees continuously demonstrate behaviors that appear to require a conscious mind: probabilistic learning [83], sophisticated spatial memory, and even what might be called “play” in object manipulation [84]. However, these apparently miraculous phenomena might emerge from surprisingly non-conscious mechanisms. The monitoring of uncertainty provides an illustrative example: when a bee “decides” not to respond to a difficult discrimination, this might appear to be metacognition, but critics suggest that this simply reflects signal detection thresholds in sensory circuits, not metacognitive awareness [83]. The symbolic communication of the waggle dance, however elegant, is genetically encoded and requires no understanding of receiver mental states [10]. Even emotion-like states, with their dopaminergic responses to rewards [42], might drive behavioral conditioning without subjective pleasure [78].

Critics invoke Morgan’s Canon [85], that principle of parsimony that reminds the scientific community that simpler explanations should precede claims of consciousness [86]. The burden of proof, they contend, lies not in demonstrating that insects possess functional complexity, but that they possess that interior dimension of “what it is like” that Nagel (1974) [87] identified as the heart of the consciousness problem. Perhaps the most profound challenge in the debate over bee consciousness resides in the epistemological limitations faced by current research. The absence of consensus on behavioral or neural correlates of consciousness in invertebrates renders the debate empirically intractable with current frameworks [11]. The risk of anti-anthropomorphism emerges when human-like experiences are projected onto bees, potentially confusing functional homology with phenomenological similarity [88].

## 8. Limitations and Future Research Directions

The evidence presented throughout this perspective article demonstrates that honeybees could possess many of the same neurocognitive and behavioral markers of sentience that justified the inclusion of cephalopods in welfare legislation. Both groups exhibit: (1) complex sensory integration capabilities; (2) advanced learning and memory functions; (3) behavioral flexibility and problem-solving; (4) emotional-like states modulated by similar neurochemical systems; (5) capacity for pain perception and suffering.

The New York Declaration on Animal Consciousness (2024) lends further weight to these findings, affirming that conscious experience likely exists along a continuum that transcends traditional vertebrate–invertebrate divides. Recent work by Formato et al. (2024) in *Frontiers in Animal Science* further consolidates this position, providing a comprehensive framework for defining and implementing honeybee welfare standards that align with their demonstrated cognitive capacities [22]. However, translating this ethical imperative into practical beekeeping standards presents unique challenges—from the economic impact on small-scale apiculturists to the technical difficulties of monitoring welfare in complex superorganisms.

While legislative recognition of bee welfare is scientifically justified, implementation must minimize burdens on beekeepers. Welfare improvements can enhance hive productivity and honey quality, but it is mandatory to propose gradual adoption of the proposed protocols and economic incentives, specifically, EU subsidies for certified welfare-friendly beekeeping (analogous to organic farming). Phased implementation and financial incentives (e.g., certification premiums, subsidies) can mitigate burdens on beekeepers. Pilot programs could identify cost-effective protocols. While welfare protocols (e.g., transport limits, non-lethal disease monitoring) may initially increase operational costs for beekeepers, evidence from vertebrate farming suggests that reduced stress improves colony health and productivity, offsetting long-term expenses. Economic tools—such as EU subsidies for welfare certification or market premiums for high-welfare honey—could ease this transition, particularly for small-scale apiculturists.

However, in light of the considerations raised by critics of sentience, it is necessary to state that this manuscript is not intended as a conclusive statement, but as part of a broad comparative and probabilistic investigation. Furthermore, we emphasize the importance of avoiding any overinterpretation of research results, while continuing to support precautionary ethical considerations in line with current concerns about well-being and the environment.

Finally, it is important to emphasize that, in our opinion, if the evidence brought to attention leads to a debate on the issue, it is important that beekeeping research is not hindered by excessive bureaucracy. The sector is facing a deep crisis due to numerous threats. To achieve significant scientific advancements and innovations in this livestock sector, the work of scientists must continue to be as smooth as it is today.

Table 3 outlines actionable proposals to support this thesis, acknowledging challenges while highlighting benefits.

## 9. Conclusions

The welfare and protection of invertebrates, particularly farmed invertebrates used for production purposes such as honeybees, is an increasingly relevant topic. Given the presence of skeptical positions, the implementation of protective measures should be gradual and evidence-based, supported by the establishment of a scientific observatory tasked with continuously updating regulatory standards in response to new findings—an approach that would honor both the spirit of the New York Declaration and the practical realities of beekeeping.

These reforms would not only yield concrete economic benefits—such as improved colony health, reduced hive mortality, and more sustainable productivity, facilitating access to high-value markets—but would also rectify an ethical and legal oversight that is no longer defensible. This approach aims to reconcile scientific advancement with animal ethics and sustainable animal husbandry, in line with the One Health framework, and to meet the challenges of the 21st century with responsibility and foresight. This inclusion proposal responds to the expectations of animal welfare and sustainability-conscious consumers, who consciously choose products from welfare-friendly farms, while offering tangible economic benefits for the beekeeper.

## Figures and Tables

**Table 1:** Operational definitions of key terms.

Term	Operational Definition
Sentience	Capacity to experience subjectively valenced states (e.g., pain, pleasure), distinct from nociception (unconscious physiological response).
Emotion	Neurophysiological response to salient stimuli, measurable through behavioral shifts (e.g., cognitive biases). Not equivalent to complex human emotions.
Consciousness	Here restricted to “access consciousness”: information integration for flexible decision-making (e.g., spatial navigation).
Nociception and behavioral responses	Aversive response to harmful stimuli, with potential affective components (e.g., prolonged wound-grooming). Not identical to vertebrate pain.
Intelligence	Complex behavioral adaptation (e.g., associative learning, symbolic communication). Does not imply human-like planning or abstraction.

**Table 2:** Comparative analysis of sentience markers in honeybees and cephalopods.

Sentience Markers	Honeybees (*Apis mellifera*)	Cephalopods (e.g., *Octopus* *vulgaris*)	Legal Status in EU Legislation
Neural Architecture	1 million neurons with high synaptic density; mushroom bodies for multisensory integration	Distributed nervous system (60% neurons in arms); vertical, superior, and optic lobes	Cephalopods protected under Directive 2010/63/EU; honeybees excluded
Neural Synchronization	Alpha oscillations similar to those in vertebrates	“Low amplitude fast” neural activity functionally equivalent to mammalian thalamocortical complex	Inconsistent recognition despite similar functional properties
Associative Learning	Complex associative learning and rule-based flexibility	Tool use and problem-solving abilities	Both demonstrate advanced learning beyond simple stimulus-response
Memory Functions	Long-term memory; memory consolidation during sleep	Episodic-like memory systems	Both exhibit memory systems supporting complex behaviors
Emotional-like States	Stress-induced pessimistic bias; dopamine-mediated positive states	Stress responses and preference behaviors	Both show evidence of affective states beyond mere reactivity
Communication	Waggle dance with symbolic coding of distance and direction	Complex signaling systems including body postures and color changes	Both utilize sophisticated communication systems
Spatial Cognition	Cognitive map-like representations; novel shortcut capabilities	Navigation using landmarks and memory	Both employ advanced spatial representation systems
Abstraction	Concept formation (“same” vs. “different”)	Abstract problem solving	Both capable of abstraction beyond immediate stimuli
Individual Differences	Scout bees showing consistent risk-taking behaviors; genetic basis for personality traits	Individual behavioral differences documented	Both exhibit individual variations in behavioral traits
Pain Perception	Mechanosensory and chemosensory structures; opioid-like system; behavioral responses to injury	Nociceptive systems with behavioral indicators of pain	Inconsistent regulatory recognition despite similar evidence
Sleep Patterns	Three distinct sleep stages with rebound effects after deprivation	Rest states with homeostatic regulation	Both demonstrate complex rest behaviors beyond simple inactivity

**Table 3:** Proposed framework for integrating honeybee welfare into EU legislation.

Aspect	Current Status	Scientific Evidence	Recommended Policy Changes	Implementation Challenges	Expected Benefits
Legal Recognition	Excluded from EU Directive 2010/63/EU	Substantial evidence of sentience markers comparable to those of protected species	Formal inclusion of *Apis mellifera* in Annex I of Directive 2010/63/EU	Defining appropriate welfare criteria for superorganisms	Ethical consistency in EU animal welfare legislation
Precautionary Principle	Not applied to honeybees despite Article 191 TFEU	Growing scientific consensus on bee cognition and emotion	Apply precautionary principle in the same way as previously to cephalopods	Balancing precaution with practical apiculture needs	Protection during scientific uncertainty
Anesthetic Protocols	No standardized requirements	Evidence of pain perception and stress responses	Introduce appropriate anesthesia for invasive procedures	Developing bee- specific anesthetic methods	Reduced suffering during experimentation
Transport Standards	Limited regulation	Stress from transport impairs spatial memory and cognitive function	Develop specific transport protocols minimizing stress	Implementation costs for small-scale beekeepers	Improved colony health and reduced mortality
Handling Practices	Minimal guidelines	Chronic stress affects immune function and survival	Establish handling standards based on behavioral indicators	Training requirements for bee- keepers	Enhanced productivity and sustainability
Regulatory Oversight	Fragmented monitoring	Multiple neurobiological and behavioral markers available	Establish permanent scientific observatory for continuous updates	Coordination between research and regulatory bodies	Evidence-based policy evolution
One Health Framework	Limited integration	Links between bee welfare, ecosystem health, and food safety	Incorporate bee welfare into broader One Health policies	Cross-disciplinary coordination	Improved public health and ecosystem resilience
Economic Impact	Cost-based approach	Growing market for welfare-certified products	Phased implementation with support mechanisms	Initial adaptation costs	Access to high-value markets and consumer trust
Research Ethics	Variable standards	Comparable cognitive complexity to protected species	Standardized ethical review for bee research	Methodological adaptations in research	Increased reliability and reproducibility of research
Colony Assessment	Limited systematic monitoring	Behavioral indicators of colony well-being	Develop standardized welfare assessment tools	Practical implementation in field conditions	Early detection of welfare issues

## Data Availability

The original contributions presented in this study are included in the article. Further inquiries can be directed to the corresponding author.
